# Assessment of Spinal and Pelvic Kinematics Using Inertial Measurement Units in Clinical Subgroups of Persistent Non-Specific Low Back Pain

**DOI:** 10.3390/s24072127

**Published:** 2024-03-26

**Authors:** Liba Sheeran, Mohammad Al-Amri, Valerie Sparkes, Jennifer L. Davies

**Affiliations:** 1 School of Healthcare Sciences, Cardiff University, Cardiff CF14 4XN, UK; al-amrim@cardiff.ac.uk (M.A.-A.); sparkesv@cardiff.ac.uk (V.S.); daviesj@cardiff.ac.uk (J.L.D.); 2 Biomechanics and Bioengineering Research Centre Versus Arthritis, Cardiff University, Cardiff CF10 3AT, UK

**Keywords:** non-specific low back pain, inertial measurement unit, range of motion, kinematics, spine, pelvis, clinical movement analysis, clinical classification

## Abstract

Inertial measurement units (IMUs) offer a portable and quantitative solution for clinical movement analysis. However, their application in non-specific low back pain (NSLBP) remains underexplored. This study compared the spine and pelvis kinematics obtained from IMUs between individuals with and without NSLBP and across clinical subgroups of NSLBP. A total of 81 participants with NSLBP with flexion (FP; *n* = 38) and extension (EP; *n* = 43) motor control impairment and 26 controls (No-NSLBP) completed 10 repetitions of spine movements (flexion, extension, lateral flexion). IMUs were placed on the sacrum, fourth and second lumbar vertebrae, and seventh cervical vertebra to measure inclination at the pelvis, lower (LLx) and upper (ULx) lumbar spine, and lower cervical spine (LCx), respectively. At each location, the range of movement (ROM) was quantified as the range of IMU orientation in the primary plane of movement. The ROM was compared between NSLBP and No-NSLBP using unpaired *t*-tests and across FP-NSLBP, EP-NSLBP, and No-NSLBP subgroups using one-way ANOVA. Individuals with NSLBP exhibited a smaller ROM at the ULx (*p* = 0.005), LLx (*p* = 0.003) and LCx (*p* = 0.01) during forward flexion, smaller ROM at the LLx during extension (*p* = 0.03), and a smaller ROM at the pelvis during lateral flexion (*p* = 0.003). Those in the EP-NSLBP group had smaller ROM than those in the No-NSLBP group at LLx during forward flexion (Bonferroni-corrected *p* = 0.005), extension (*p* = 0.013), and lateral flexion (*p* = 0.038), and a smaller ROM at the pelvis during lateral flexion (*p* = 0.005). Those in the FP-NSLBP subgroup had smaller ROM than those in the No-NSLBP group at the ULx during forward flexion (*p* = 0.024). IMUs detected variations in kinematics at the trunk, lumbar spine, and pelvis among individuals with and without NSLBP and across clinical NSLBP subgroups during flexion, extension, and lateral flexion. These findings consistently point to reduced ROM in NSLBP. The identified subgroup differences highlight the potential of IMU for assessing spinal and pelvic kinematics in these clinically verified subgroups of NSLBP.

## 1. Introduction

Low back pain (LBP) is the most prevalent musculoskeletal pain disorder and a global leading cause of disability [1]. It leads to impaired activity and function and absenteeism, producing profound health and socio-economic impacts [2]. Consequently, LBP has developed into a major global public health concern [3]. A total of 90% of LBP lacks identifiable pathology and is termed ‘non-specific’ low back pain (NSLBP) [4]. NSLBP is multifactorial, with physical, psychological, cognitive, behavioural, lifestyle, and societal conditions all known to contribute to the disorder [2,5]. One proposed contributing factor is related to movement alterations in the spine and pelvis, associated with movement-related fear [5], loss of function [6], physical deconditioning [7], and subsequent disability [8]. The cornerstone of NSLBP management endorsed by clinical guidelines is therefore focused on disrupting this mechanism through education, self-management, and exercise customised to individual needs and capabilities [9,10]. However, designing customised management for NSLBP is challenging given its complexity and heterogeneity, including the diverse nature of the movement alterations observed kinematically and the varied underlying mechanisms [11,12,13].

Numerous clinical classification systems have been developed to identify NSLBP clusters or subgroups with the intention of helping clinicians better customise management [14]. The clinical classification process involves a thorough subjective and physical assessment to establish the dominant physical, behavioural, lifestyle and psycho-social drivers underlying the pain disorder [15,16,17]. The clinical physical examination consists of visually assessing spinal function across various movement planes while sitting, standing, and lying down. It is limited to subjective estimates of movement visible to the human eye such as gross range of movement, noticeable changes in speed of movement and associated pain responses [18,19]. 

Objective comparisons of spinal and pelvic kinematics have been performed in laboratory settings and have identified distinct variations in clinical subgroups of NSLBP in the upper and lower lumbar spine and the pelvis during various functional movement tasks [20,21,22,23,24]. The cost and expertise required for laboratory movement analysis, however, prohibit this level of assessment in clinical settings at scale. A method that allows for objective and accurate movement analysis of spinal and pelvic movements away from the laboratory setting is therefore of clinical value. It would facilitate the delivery of highly personalised management both through offering accurate assessment, progress tracking, as well as impairment- and capability- specific exercise feedback [25]. This is of relevance considering the current shift in focus within multinational clinical guidelines for management of musculoskeletal pain conditions towards technologically supported personalised interventions [26].

Inertial measurement units (IMUs) are a portable and inexpensive alternative to laboratory-based motion capture systems that have been shown to give valid and reliable kinematic data for the lower limbs when used by a clinician [27]. There is also growing research exploring the utility of IMUs for movement analysis of the spine and pelvis [28,29,30,31,32], including in LBP [33,34,35,36,37,38,39,40,41,42,43,44,45,46,47,48,49]. To date, however, only one group other than ourselves has used IMUs to study spinal and pelvic movement in subgroups of individuals with LBP [50]. In this study, IMUs positioned at T12 and S2 assessed lumbar, trunk, and pelvic kinematics during forward flexion and detected four distinct movement impairment types typically (though not exclusively) associated with LBP [50]. These results indicate promise in the ability of IMUs to detect variations in spinal and pelvic movement patterns linked to LBP; however, the observed subgroups were not verified in an independent sample. This raises the possibility that the detected clusters may be specific to the sample rather than representative of the broader LBP population [50]. In addition, this study evaluated lumbar spine as a single segment and only during forward flexion [50]. Contemporary clinical classification models recognise that distinct movement impairments associated with LBP occur at the upper and lower lumbar spine and the pelvis and in various movement directions [23,24,51], triggering distinct pain responses and impacting movement across all planes [15]. Studying the utility of IMUs in detecting kinematic differences among clinically verified NSLBP subgroups in different movement directions and at different spine and pelvic levels is therefore essential for devising clinically useful targeted, movement-based rehabilitation interventions.

The crucial, yet unanswered, question is whether IMUs can detect distinct alterations in movements in multiple planes in individuals with NSLBP belonging to clinically verified NSLBP subgroups derived from an evidence-based clinical classification process. Our preliminary analysis revealed that angular inclinations of the trunk and pelvis, obtained from IMUs at various levels of the spine and pelvis and inputted into a machine learning model, can accurately discriminate between those with and without NSLBP, as well as among clinically verified clusters of NSLBP [52].

The purpose of this study was to compare the angular inclination of the trunk and pelvis obtained from multilevel IMUs during movements across multiple planes between (i) individuals with and without NSLBP and (ii) individuals belonging to two clinical motor control impairment subgroups of NSLBP (flexion and extension patterns).

## 2. Materials and Methods

A cross-sectional case-control study design was deployed to compare kinematics of individuals with and without NSLBP and across two clinically verified subgroups of NSLBP. A preliminary analysis of these data was reported previously [52].

### 2.1. Participants

Eighty-six participants with persistent NSLBP were recruited from Cardiff and Vale Orthopaedic Centre waiting lists for physiotherapy and staff members at Cardiff University with self-reported LBP. Twenty-six controls without LBP were recruited from staff members at Cardiff and Vale University Health Board and Cardiff University. All participants attended a single experimental session at the Research Centre for Clinical Kinaesiology at the School of Healthcare Sciences, Cardiff University. Each participant provided written informed consent prior to inclusion in the study as per the Biomechanics and Bioengineering Research Centre Versus Arthritis Research Protocol, which was reviewed and approved by the Health and Care Research Wales ethics committee, Wales REC 3 (reference 10/MRE09/28, IRAS project number 51853).

All potential participants were first screened for eligibility over the telephone and then in person, where an experienced physiotherapy clinician (LS) assessed for inclusion and exclusion criteria (Table 1). The direction of the motor control impairment (either flexion or extension) in the recruited participants with NSLBP was then confirmed by a second physiotherapist (VS) with expertise in classification of NSLBP using the clinical multidimensional classification (MDC) model described in detail elsewhere [15], with conflicts resolved by a third expert.

All selected participants were then invited to a data collection visit where height and weight were collected. In addition, prior to performing any physical tasks, participants with NSLBP completed questionnaires to characterise their level of pain, disability, and fear avoidance. Level of pain was evaluated using a visual analogue scale (VAS), with numbers zero to ten displayed at equal intervals along the line and anchored with “No pain” at zero and “Worst possible pain” at ten [53]. Participants were asked to select a number that best represented the level of pain intensity that they were experiencing ‘right now’, ‘typical/average pain’, ‘pain at its best’, and ‘at its worst’. The four VAS ratings were then averaged to obtain a level of pain. Level of disability was evaluated using the Oswestry disability index (ODI), a reliable and valid measure in LBP populations [54]. ODI scores can range from zero to 100%, with higher scores indicating more severe disability. Fear avoidance was evaluated using the Tampa Scale of Kinesiophobia, a validated fear of movement and avoidance measure in people with LBP [55]. The Tampa Scale of Kinesiophobia score can range from 17 to 68, with higher scores indicating a greater degree of kinesiophobia.

### 2.2. IMU Configuration 

**Figure 1:**
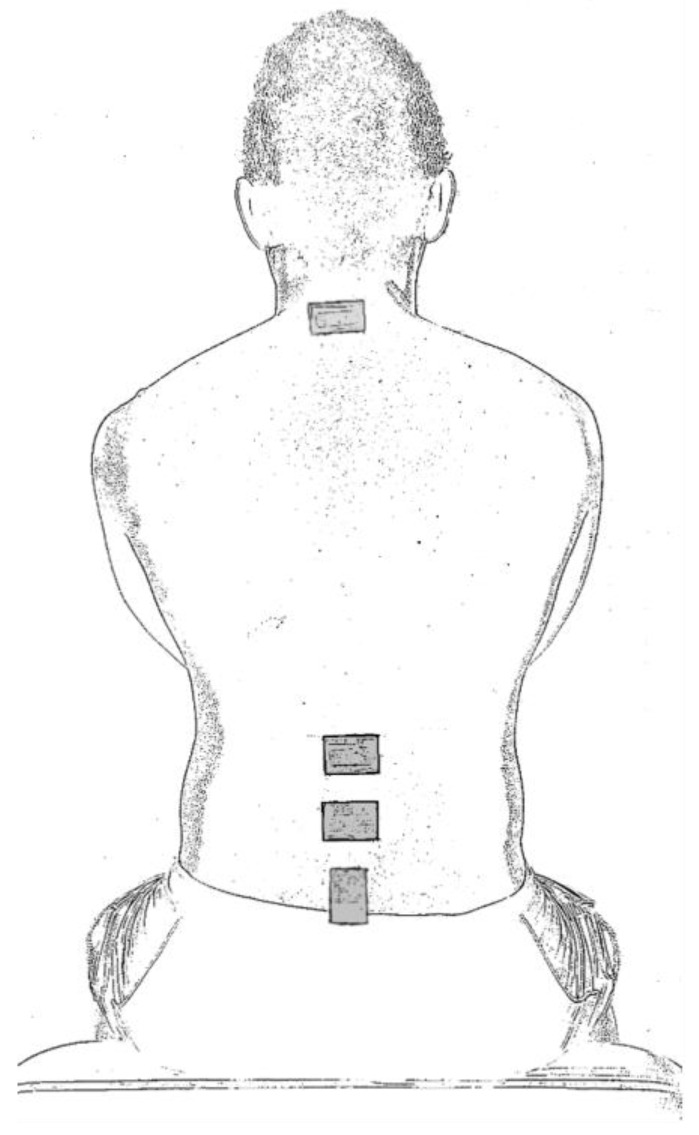
Positioning of inertial measurement units.

IMUs (MTw2 trackers; Xsens Technologies B.V., Enschede, The Netherlands) were placed over the spinous processes of the seventh cervical vertebra (C7) second and fourth lumbar vertebra (L2 and L4) and sacrum (S2) to measure orientation at the lower cervical spine, upper lumbar spine, lower lumbar spine, and pelvis, respectively. The sacrum IMU was placed vertically to accommodate the retroreflective markers placed over the sacrum as part of separate data collection. IMU positions are shown in Figure 1. 

### 2.3. Testing Protocol

Participants performed three routine clinical assessment tests that have been deemed clinically important for assessment of NSLBP by expert consensus [56]: spine flexion, extension, and lateral flexion. For each task, participants received basic instructions on how to perform the test (Table 2), with no further instruction on the method or speed of the movement. To ensure safety, participants performed two practice repetitions of each test, followed by performing 10 repetitions of the test. 

### 2.4. Data Acquisition and Processing

For the 86 participants with NSLBP enrolled, 1 was excluded on the day of the data collection reporting acute LBP on arrival, rendering them unable to safely undertake the testing procedure. A complete dataset (minimum 50% of total 10 repetitions of each movement) was obtained from 81 participants and included in the analyses. For the first 20 LBP and 11 control participants, IMU data were collected using MVN Analyse software (version 3, Xsens Technologies B.V., Enschede, The Netherlands)) at 60 Hz. For all subsequent participants (65 LBP, 15 control), IMU data were collected using MT manager software (version 4.3.1, Xsens Technologies B.V.) at 100 Hz. These sampling frequencies were sufficient to capture the movements performed. All IMU data were stored for offline analysis. 

All analyses were performed in Matlab (version R2019-R2022, MathWorks, Natick, MA, USA) using custom written code. For data collected with MT manager software, IMU orientation in the global frame was exported in Euler angles as a *.txt file and imported directly into Matlab. Data collected with MVN software were exported as a *.mvnx file, and IMU orientation in the global frame was extracted from this file and converted from quaternion to Euler angles in Matlab using the ‘quat2angle’ function with the rotation sequence ‘Z, Y, X’. For all IMUs, rotation around Z refers to movement in the frontal plane. For the IMU on the pelvis, rotation around Y referred to movement in the sagittal plane and X to the transverse plane. For all other sensors, rotation around Y referred to movement in the transverse plane and X referred to that in the frontal plane. Subsequently, all processing of IMU orientation in the global frame was the same for all participants.

IMU orientation was low-pass-filtered at 6 Hz using a fourth-order Butterworth filter. The start and end of each movement cycle were identified using IMU orientation in the primary plane of movement. For each IMU (pelvis, lower lumbar spine, upper lumbar spine, and lower cervical spine), the range of movement (ROM) was quantified for each movement cycle as the range of IMU orientation in the primary plane of movement. ROM therefore represents the range of IMU inclination at that location. For each participant, the ROM for each task was averaged across all movement cycles. 

### 2.5. Statistical Analysis 

Participant characteristics were compared across three participant groups (flexion pattern (FP) NSLBP, extension pattern (EP) NSLBP, no NSLBP) using χ^2^ test for sex and one-way analysis of variance (ANOVA) for age and body mass index and were compared across the two NSLBP groups using independent *t* tests for VAS pain intensity score, ODI score, and Tampa Scale of Kinesiophobia score. 

At each location (pelvis, lower lumbar spine, upper lumbar spine, lower cervical spine), ROM during flexion, extension, and lateral flexion was compared across participants with and without LBP using an independent *t* test. Significance level (alpha) was set at *p* ≤ 0.05 given the exploratory, rather than hypothesis-testing, nature of the study. In a further subgroup analysis, ROM was also compared across the three participant groups (FP-NSLBP, EP-NSLBP, and no NSLBP) using a one-way ANOVA. All significant main effects in ANOVA (*p* ≤ 0.05) were followed up with pairwise post hoc *t* tests with Bonferroni correction for multiple comparisons within each variable. Statistical analyses were performed in Matlab.

## 3. Results

### 3.1. Participant Characteristics

All participants belonged to the white British ethnic group and completed the data collection without any adverse effects. Of the 86 NSLBP participants enrolled, 1 was excluded on the day of the data collection reporting acute LBP on arrival, rendering them unable to safely undertake the testing procedure. A further four participants with incomplete data with single repetition of movements recorded were excluded from the analyses. Of the 81 LBP participants, 38 had characteristics of FP-NSLBP and 43 had those of EP-NSLBP. The final sample participant characteristics are shown in Table 3. 

### 3.2. Between-Group Differences in Spinal and Pelvic Kinematics

**Figure 2:**
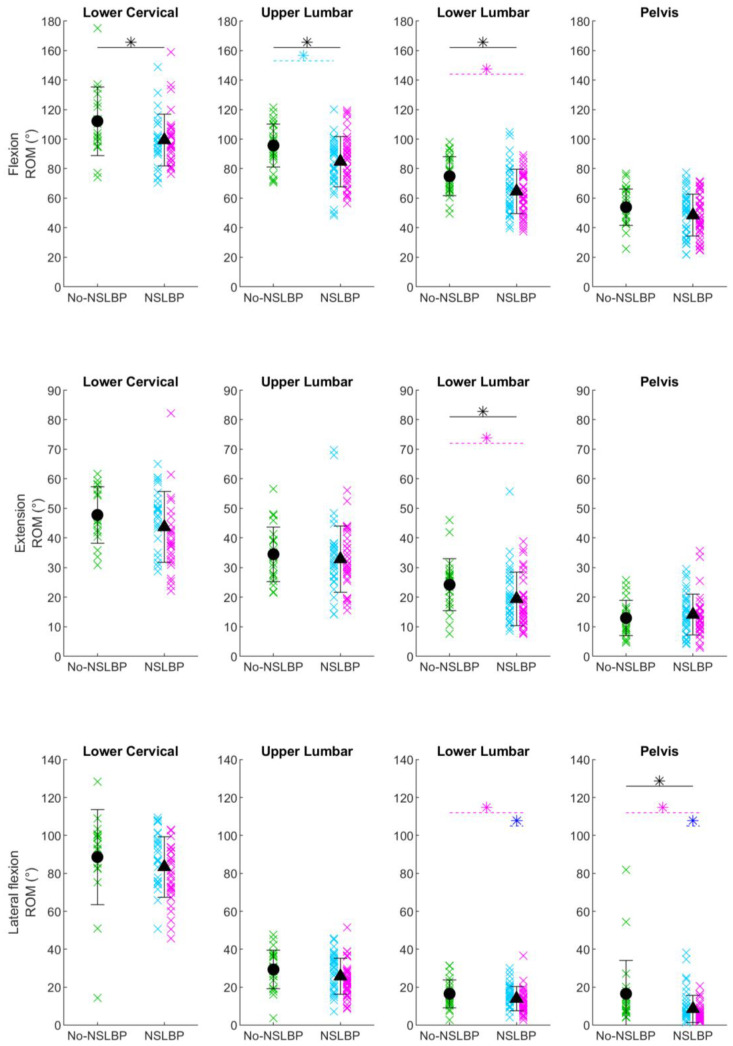
Range of motion. Range of motion (ROM) of IMU inclination at the lower cervical spine (seventh cervical vertebra), upper lumbar spine (second lumbar vertebra), lower lumbar spine (fourth lumbar vertebra), and pelvis (sacrum) for each participant without non-specific low back pain (No-NSLBP; green crosses), with flexor-pattern non-specific low back pain (FP-NSLBP; cyan crosses), and with extensor-pattern non-specific low back pain (EP-NSLBP; magenta crosses) performing forward flexion (**top row**), extension (**middle row**), and lateral flexion (**bottom row**) of the spine. Circles indicate mean of participants with No-NSLBP. Triangles indicate mean of participants with NSLBP. Black asterisks indicate significant differences between patients with No-NSLBP and NSLBP (*p* ≤ 0.05, independent *t* test). Cyan asterisks indicate significant post hoc pairwise differences between FP-NSLBP and No-NSLBP subgroups. Magenta asterisks indicate significant post hoc pairwise differences between EP-NSLBP and No-NSLBP subgroups. Blue asterisks indicate significant post hoc pairwise differences between EP-NSLBP and FP-NSLBP subgroups.

The ROMs of IMU inclination at each location for NSLBP and no-NSLBP, as well as NSLBP subgroups, are shown in Figure 2 and Table 4.

#### 3.2.1. NSLBP vs. no-NSLBP

During forward flexion, the NSLBP group had a smaller ROM at the lower cervical spine (*p* = 0.01) and at the upper (*p* = 0.005) and lower (*p* = 0.003) lumbar spine compared to the no-NSLBP group (black asterisks in Figure 2). During extension, the NSLBP group had a smaller ROM at the lower lumbar spine (*p* = 0.03); during lateral flexion, the NSLBP group had a smaller ROM at the pelvis (*p* = 0.003; black asterisk in Figure 2). 

#### 3.2.2. No-NSLBP vs. EP-NSLBP vs. FP-NSLBP 

During forward flexion, there was a main effect of group for ROM at the lower cervical spine (*p* = 0.05) and the upper (*p* = 0.02) and lower (*p* = 0.008) lumbar spine. At the lower cervical spine, no pairwise post hoc comparisons were significant, but the tendency was for both NSLBP subgroups to have a smaller ROM than those in the No-NSLBP subgroup. At the upper lumbar spine, the FP-NSLBP subgroup had a smaller ROM than the No-NSLBP group (Bonferroni-corrected post hoc *p* = 0.02; cyan asterisk in Figure 2); at the lower lumbar spine, the EP-NSLBP subgroup had a smaller ROM than the No-NSLBP group (Bonferroni-corrected post hoc *p* = 0.008; magenta asterisk in Figure 2) 

During extension, there was a main effect of group for ROM at the lower cervical spine (*p* = 0.04) and at the lower lumbar spine (*p* = 0.05). At the lower cervical spine, no pairwise post hoc comparisons were significant, but the tendency was for EP-NSLBP to have a smaller ROM. At the lower lumbar spine, the EP-NSLBP subgroup had a smaller ROM than the No-NSLBP group (Bonferroni-corrected post hoc *p* = 0.04; magenta asterisk in Figure 2). 

During lateral flexion, there was a main effect of group for ROM at the upper (*p* = 0.04) and lower (*p* = 0.01) lumbar spine and the pelvis (*p* = 0.003). At the upper lumbar spine, no pairwise post hoc comparisons were significant, but the tendency was for EP-NSLBP to have a smaller ROM. At the lower lumbar spine and the pelvis, the EP-NSLBP subgroup had a smaller ROM than the no-NSLBP group (Bonferroni-corrected post hoc *p* = 0.04 and 0.005, respectively) and a smaller ROM than the FP-NSLBP group (Bonferroni-corrected post hoc *p* = 0.02 and 0.04 respectively; magenta and blue asterisks in Figure 2). 

## 4. Discussion

This study pioneered the use of IMUs to comprehensively analyse spinal and pelvic movements in individuals with NSLBP belonging to clinically verified subgroups derived from an evidence-based clinical classification process. Previous research used IMUs to assess lumbar spine as a single segment, limiting our current understanding of NSLBP complexities. Our study progressed this evaluation by broadly aligning with contemporary classification models of NSLBP, which recognise distinct variations in regional spinal and pelvic kinematics across movements in multiple planes [23,24,51]. The findings identified the utility of IMUs in detecting specific kinematic patterns within NSLBP subgroups, offering invaluable insights for tailoring personalised rehabilitation interventions. Furthermore, this study emphasises that while a single IMU placed at the top of the spine (C7) can effectively quantify gross flexion ROM deficits, multiple IMUs are necessary to reveal movement variations across the clinical NSLBP subgroups. This research has practical implications for clinicians seeking to optimise NSLBP management in accordance with clinical guidelines promoting approaches customised to individual needs and capabilities [10].

### 4.1. Trunk Kinematics 

The inclination in the global frame of the single IMU placed over C7 reflects the combined inclination of every segment from the shanks upward. Participants were instructed to ‘bend their back’ and did not exhibit visible movement at the ankle or knee joints. The ROM of the IMU over C7 may therefore be broadly interpreted as representing the ROM of the trunk and pelvis. Significant differences in the ROM at this location were observed only during spine flexion, where individuals with NSLBP exhibited a smaller ROM than those without NSLBP. These findings align with those of earlier research that identified trunk flexion as capable of discriminating between individuals with and without LBP [57]. However, in contrast to Waddell et al. [58], we observed no differences in this proxy of trunk movement in extension. In the remaining tasks, no other differences in ROM at this location were observed, either in general (NSLBP vs. No-NSLBP) or across subgroups (no-NSLBP vs. EP-NSLBP vs. FP-NSLBP), which is consistent with the findings of previous research [21,58].

Further in support of these results is a recent systematic review which concluded that, out of the multitude of movements kinematically evaluated in research, spinal flexion appears to be the most sensitive and relevant movement test for detecting kinematic differences between individuals with and without LBP regardless of the instrument used [59]. While uncertainty persists regarding the role of trunk kinematics in detecting NSLBP subgroup differences, there may still be clinical value in quantifying a gross measure of flexion as an NSLBP biomarker that could be used to monitor progress of individuals undergoing treatment and ensuring the treatment plans evolve based on response and changing needs. Our results demonstrate that this may be effectively achieved using a single IMU.

### 4.2. Upper and Lower Lumbar Spine and Pelvic Kinematics 

Compared to the asymptomatic group, individuals with NSLBP demonstrated reduced ROM at the upper and lower lumbar spine during flexion and at the lower lumbar spine during extension. These findings agree with those of two recently published systematic reviews and meta-analyses demonstrating sagittal plane ROM limitations in the lumbar spine in LBP evaluated using optoelectronic, electromagnetic, IMU, and other measurement systems [60,61]. Our study further contributes to the field by highlighting that reduced ROM during flexion is evident at both the upper and lower lumbar spine regions, whereas differences in extension between those with and without NSLBP are primarily observed at the lower lumbar spine region.

Our subgroup analysis revealed spinal movement patterns consistent with the previously described characteristics for each subgroup [15]. Specifically, the EP-NSLBP subgroup exhibited smaller ROM at the lower lumbar spine during flexion and extension compared to the asymptomatic group, whereas the FP-NSLBP group had comparable ROM at the lower lumbar spine but smaller ROM at the upper lumbar spine compared to the No-NSLBP and EP-NSLBP groups during flexion. These results align with observations from Dankaerts et al. [37], who assessed similar subgroups and found that during flexion, participants with EP-NSLBP moved with an extended spine, while participants with FP-NSLBP achieved comparable lower lumbar flexion to the no-LBP counterparts. In contrast to our findings, a previous study by Dankaerts et al. [22] reported that, during sitting, individuals with FP-NSLBP had a more flexed upper lumbar spine curvature compared to asymptomatic individuals. Methodological discrepancies may have contributed to the contrasting findings. In addition, the repetitive execution of a forward bend in our study, as opposed to sitting, may have presented an increased challenge, potentially triggering pain-related fear and a subsequent protective response, previously found to be predictive of lumbar ROM deficit in people with chronic low back pain during a lifting task [62].

At the pelvis, our results indicate a significantly reduced frontal plane ROM during lateral flexion among individuals in the NSLBP group, with no other differences observed between those with and without NSLBP. This finding of no difference in ROM during forward flexion contrasts that of a study by Laird et al. (2019), which reported significant differences only in pelvic flexion during forward spine bend [48]. Upon closer examination, however, our study revealed ROM at the pelvis during flexion was 54 degrees in the No-NSLBP group (*n* = 26) and 48 degrees in the NSLBP group (*n* = 81), and these values are comparable to those reported by Laird et al. (2019), which were 59 degrees (*n* = 124) and 48 degrees (*n* = 140), in LBP vs. no-LBP, respectively. The overall pattern of results is therefore similar, and any statistical discrepancies between the studies may be attributed to the smaller sample size in our study.

Interestingly, our subgroup analyses reveal that the differences observed in ROM at the pelvis during lateral flexion were primarily influenced by the EP-NSLBP subgroup, which also displayed a smaller ROM at the lower lumbar spine during lateral flexion. To the best of our knowledge, there have been no previous studies investigating frontal plane pelvic kinematics in specific subsets of individuals with LBP. However, there is compelling evidence indicating that limitations in lateral flexion movement are predictive of the development of LBP [63]. Our study suggests that the reduction in lateral flexion is potentially more strongly associated with the EP-NSLBP subgroup, but this hypothesis awaits confirmation through future research studies.

### 4.3. Clinical Implications

This study has important implications for clinical practice. It demonstrates the practicality of employing IMUs to identify variances in movement patterns among individuals with NSLBP relative to those without pain. Furthermore, our study is first in using IMUs to differentiate between clinically verified subgroups of NSLBP derived from an evidence-based clinical classification process. Our findings expand upon and substantiate the conclusions of prior research primarily focused on forward bending [48,49,50], broadening the scope to encompass assessments of movements in multiple directions. Notably, our research represents a pioneering effort in utilising absolute angles from single IMUs to study variations across clinically verified NSLBP subgroups. This approach reduces the need for complex data processing, thus opening potential opportunities for clinical utility, including simple remote monitoring.

This study revealed that IMUs can detect distinct movement patterns within clinically verified NSLBP subgroups, potentially assisting clinicians in assessment and tailoring rehabilitation. Specifically, clinicians may consider that individuals with FP-NSLBP may demonstrate deficits in ROM at the upper lumbar spine ROM in forward flexion, while individuals with EP-NSLBP may exhibit diminished ROM at the lower lumbar spine during forward flexion, extension, and lateral flexion, along with reduced lateral flexion at the pelvis.

Finally, building on our preliminary analysis [52], this study highlights the potential of utilising absolute IMU angles to train machine learning models for accurate NSLBP classification. These insights have significant potential for advancing technology-supported personalised self-management interventions, modernising the design and delivery of personalised management to meet each patient’s needs in a timely and responsive manner. 

### 4.4. Limitations 

Several limitations should be considered when interpreting our results. Participants with NSLBP were, on average, older and had a higher BMI than asymptomatic participants, and sex distribution differed across the subgroups (FP-NSLBP: 66% male, EP-NSLBP: 74% female). Although statistically different, a closer inspection revealed similar age and BMI category distributions across the subgroups, suggesting a limited impact on the overall results. However, the subgroup difference in sex distribution must be acknowledged as a potential confounder, particularly considering the distinct impairments prevalence among the different sexes shown in previous MCI cohort comparisons [20,22,23]. Future NSLBP MCI classification research should consider the influence of sex distribution on lumbo-pelvic kinematics across subgroups. 

Unique to skin-mounted measurement systems, excess adipose tissue may introduce motion artifacts due to skin and subcutaneous tissue vibration. Given the slow-motion nature of the tasks assessed in this study, the impact of excess tissue is unlikely to be practically significant. To minimise potential effects, IMUs were affixed over bony landmarks of the spine and pelvis parts, with continuous position and affixation checks during data collection. 

Finally, recognising the intricate nature of NSLBP, being influenced by various interacting physical, psychological, behavioural, and lifestyle factors, individuals with NSLBP consistently exhibit movement deficits [48,61]. Our research shows that IMUs can detect patterns in these deficits within clinically verified NSLBP subgroups, suggesting the potential for IMUs by aiding clinicians in detecting and monitoring subgroup-specific deficits and personalising rehabilitation for improved clinical outcomes.

### 4.5. Future Research 

This study primarily aimed to assess the capability of IMUs in discerning kinematic disparities in the spine and pelvis without considering other factors such as movement speed and coordination. Although movement speed and coordination were used to subgroup LBP [50], it remains uncertain whether these factors vary among clinically verified subgroups of NSLBP derived from evidence-based clinical classification models. This prompts potential future investigations into the utility of IMUs for detecting clinical subgroup differences by incorporating movement speed and coordination as a feature. 

The overarching objective of LBP classification research is to facilitate targeted movement-based rehabilitation, aligning with existing clinical guidelines that advocate NSLBP management customised to meet the needs and capabilities of each individual [10]. Future endeavours should progress towards this goal, focusing on the development of targeted interventions to address distinct movement deficits and assessing whether personalising interventions in this way yields superior outcomes compared to non-targeted approaches. At present, there is a paucity of high-quality evidence regarding whether movement-based classification results in improved clinical outcomes compared to standard care [64]. Consequently, further investigation in this realm is essential.

## 5. Conclusions

This study presented the first comparative analysis of IMU-derived spine and pelvic kinematics in individuals with NSLBP within clinically verified subgroups derived from an evidence-based clinical classification process. The investigation primarily focused on comparing the inclination of the trunk and pelvis obtained from multilevel IMUs during movements across two planes, between individuals with and without NSLBP, as well as those belonging to two clinical motor control impairment subgroups of NSLBP (flexion and extension patterns).

Significant differences were observed in the ROM of the IMUs placed at the lower cervical, upper and lower lumbar spine, and pelvis between individuals with and without NSLBP, and across clinical subgroups of NSLBP, during flexion, extension, and lateral flexion. These differences consistently indicated a trend of reduced ROM in individuals with NSLBP. Notably, the extent and location of ROM deficits varied among the subgroups, suggesting the potential utility of IMUs for assessing spinal kinematics in clinically verified NSLBP subgroups. Such assessments could assist in tailoring rehabilitation approaches and monitoring the effectiveness of treatments. 

A single IMU placed over the seventh cervical vertebra may suffice for quantifying gross ROM deficits associated with NSLBP but may lack the granularity needed to characterise variations across the clinical subgroups of NSLBP. These subgroups may necessitate distinct rehabilitation protocols, and a more comprehensive approach may be required for precise assessment and management.

## Figures and Tables

**Table 1:** Eligibility criteria for participants with NSLBP.

Inclusion Criteria	
	Age between 18 and 75 years
	Low back pain of non-specific nature for longer than 3 months
	Clear mechanical basis for the pain disorder with a diagnosis of motor control impairment (MCI), characterised by unrestricted yet painful movement and pain-provoking behaviour in flexion or extension [15]. Flexion pattern (MCI-FP)—pain-provoking postures and movements involving lumbar flexion (e.g., slouched sitting, driving, bending) and pain is eased with lumbar extension postures/movements (e.g., supported/lordotic sitting, standing, walking, bending backwards) Extension pattern (MCI-EP)—pain-provoking postures and movements involving lumbar extension (e.g., standing, walking, bending backwards), and pain is eased with lumbar flexion (e.g., slouched sitting, bending forwards)
	Able to speak and understand English well enough to complete questionnaires independently
**Exclusion Criteria**	
	Primary pain area different to lower back (from T12 to buttock line), e.g., leg pain, thoracic pain
	Acute exacerbation of pain at the time of testing, rendering the individual unable to undertake the testing procedure
	Specific diagnosis for pain (nerve root compression, radicular pain/radiculopathy, disc herniation, spondylolisthesis, spinal stenosis)
	Surgery (lower limb or abdominal surgery in last 6 months, any spinal surgery)
	Injection therapy for pain relief in the last 3 months
	Rheumatologic/inflammatory disease (e.g., psoriatic arthritis, rheumatoid arthritis, ankylosing spondylitis, Scheuermann’s disease), scoliosis (if a primary pain driver)
	Progressive neurological or neurodegenerative conditions (e.g., multiple sclerosis, Parkinson’s disease, motor neuron disease)
	Red flags/serious pathology (malignancy, acute trauma such as fracture, systemic infection, spinal cord compression, cauda equina syndrome)
	Pregnancy/breast feeding

**Table 2:** Instructions given to participants to perform the clinical assessment tests.

Assessment Test	Instruction
Flexion	From standing, bend your back forwards as far as is comfortable and return up to standing
Extension	From standing, bend your back backwards as far as is comfortable and return up to standing
Lateral flexion	From standing, bend sideways as far as is comfortable to the right/left, then back to the middle and sideways to the other side and back to the middle again

**Table 3:** Participant characteristics.

	No NSLBP *n* = 26	FP-NSLBP *n* = 38	EP-NSLBP *n* = 43	*p* Value
Female	16 (62%)	13 (34%)	32 (74%) ^†^	0.001
Male	10 (38%)	25 (66%)	11 (26%) ^†^	
Age	37.0 (12.2) [22–69]	44.9 (12.3) [22–79] *	46.6 (11.6) [25–698] *	0.006
BMI (kg/m^2^)	24.7 (3.9) [19.0–34.4]	27.2 (4.3) [18.9–37.6]	28.4 (5.2) [19.8–37.3] *	0.006
VAS	-	4.0 (1.3) [1.8–6.3]	3.9 (1.2) [1–6.8]	0.76
ODI score	-	19.6 (1.6) [2–44]	19.3 (8.9) [4–37]	0.88
TSK score	-	33.2 (6.5) [17–44]	33.0 (6.9) [17–45]	0.91

Data are mean (SD) [range] or number (percentage). NSLBP, non-specific low back pain; FP, flexion pattern; EP, extension pattern; BMI, body mass index; VAS, visual analogue scale; ODI, Oswestry disability index; TSK, Tampa Scale of Kinesiophobia; * significantly different from no-NSLBP; ^†^ significantly different from FP-NSLBP.

**Table 4:** Range of motion of the IMU at the lower cervical spine, upper lumbar spine, lower lumbar spine, and pelvis for all tasks and groups.

Task	Region	No-NSLBP	NSLBP (All)	FP-NSLBP	EP-NSLBP	*t*-Test *p* Value	ANOVA Main Effect *p* Value	ANOVA Post Hoc Pairwise Comparisons (*p* Value)		
								No-NSLBP v FP-NSLBP	No-NSLBP v EP-NSLBP	NSLBP-FP v NSLBP-EP
**Flexion** **ROM (°)**	**LCx**	112 (23) [102, 123]	99 (18) [95, 104]	99 (18) [93, 106]	100 (18) [93, 106]	**0.01**	**0.05**	0.11	0.10	>0.99
	**ULx**	96 (14) [90, 101]	85 (17) [81, 89]	84 (17) [78, 89]	86 (17) [81, 91]	**0.005**	**0.02**	**0.02**	0.06	>0.99
	**LLx**	75 (13) [70, 80]	65 (15) [61, 68]	66 (16) [61, 71]	63 (14) [59, 68]	**0.003**	**0.008**	0.08	**0.005**	>0.99
	**P**	54 (12) [49, 59]	48 (14) [45, 52]	49 (15) [44, 54]	48 (13) [43, 52]	0.09	0.21	n/a		
Extension ROM (°)	LCx	48 (10) [43, 52]	44 (12) [40, 47]	47 (10) [43, 51]	40 (13) [35, 45]	0.21	0.04	>0.99	0.14	0.11
	ULx	34 (9) [30, 38]	33 (11) [30, 36]	33 (13) [29, 38]	33 (10) [29, 36]	0.53	0.80	n/a		
	LLx	24 (9) [21, 28]	19 (9) [17, 22]	21 (9) [17, 24]	18 (9) [15, 21]	0.03	0.05	0.48	0.04	0.87
	P	13 (6) [10, 15]	14 (7) [12, 16]	14 (7) [12, 17]	14 (7) [11, 16]	0.47	0.71	n/a		
**Lateral** **Flexion** **ROM (°)**	**LCx**	89 (25) [76, 101]	83 (16) [79, 88]	89 (15) [83, 95]	79 (15) [73, 85]	0.31	0.08	n/a		
	**ULx**	29 (10) [25, 33]	26 (9) [24, 28]	28 (10) [25, 31]	24 (9) [21, 26]	0.13	**0.04**	>0.99	0.07	0.11
	**LLx**	16 (7) [13, 19]	14 (6) [12, 15]	16 (6) [14, 18]	12 (6) [10, 14]	0.11	**0.01**	>0.99	**0.04**	**0.02**
	**P**	17 (18) [9, 24]	9 (7) [7, 10]	11 (9) [8, 14]	7 (4) [5, 8]	**0.003**	**0.003**	0.39	**0.005**	**0.04**

Values are mean (SD) [95% CI]. *p* values in bold are ≤0.05. If there was a significant (*p* ≤ 0.05) main effect of group in the ANOVA, post hoc pairwise comparisons were performed. *p* values for the post hoc pairwise comparisons were Bonferroni-corrected for multiple comparisons. The adjusted *p* values are presented in the table. Adjusted *p* ≤ 0.05 was considered significant. NSLBP, non-specific low back pain; FP, flexion pattern; EP, extension pattern. LCx, lower cervical spine; ULx, upper lumbar spine; LLx, lower lumbar spine; *p*, pelvis n/a indicates that no post-hoc tests were performed, due to there being no significant main effect.

## Data Availability

Data supporting the reported results can be found on the Open Science Framework—https://osf.io/fn984, accessed on 14 March 2024.

## References

[1] Hoy D., March L., Brooks P., Blyth F., Woolf A., Bain C., Williams G., Smith E., Vos T., Barendregt J. (2014). The global burden of low back pain: Estimates from the Global Burden of Disease 2010 study. Ann. Rheum. Dis..

[2] Clark S., Horton R. (2018). Low back pain: A major global challenge. Lancet.

[3] Buchbinder R., van Tulder M., Öberg B., Costa L.M., Woolf A., Schoene M., Croft P., Hartvigsen J., Cherkin D., Foster N.E. (2018). Low back pain: A call for action. Lancet.

[4] Maher C., Underwood M., Buchbinder R. (2017). Non-specific low back pain. Lancet.

[5] Osumi M., Sumitani M., Otake Y., Nishigami T., Mibu A., Nishi Y., Imai R., Sato G., Nagakura Y., Morioka S. (2019). Kinesiophobia modulates lumbar movements in people with chronic low back pain: A kinematic analysis of lumbar bending and returning movement. Eur. Spine J..

[6] Panhale V.P., Gurav R.S., Nahar S.K. (2016). Association of Physical Performance and Fear-Avoidance Beliefs in Adults with Chronic Low Back Pain. Ann. Med. Health Sci. Res..

[7] Lee J., Park S. (2017). The relationship between physical capacity and fear avoidance beliefs in patients with chronic low back pain. J. Phys. Ther. Sci..

[8] Vlaeyen J.W.S., Linton S.J. (2000). Fear-avoidance and its consequences in chronic musculoskeletal pain: A state of the art. Pain.

[9] Oliveira C.B., Maher C.G., Pinto R.Z., Traeger A.C., Lin C.-W.C., Chenot J.-F., van Tulder M., Koes B.W. (2018). Clinical practice guidelines for the management of non-specific low back pain in primary care: An updated overview. Eur. Spine J..

[10] NICE (2016). National Institute of Health and Care Excellence, Low Back Pain and Sciatica over 16s: Assessment and Management (NICE Guideline NG59).

[11] van Dieën J.H., Reeves N.P., Kawchuk G., van Dillen L.R., Hodges P.W. (2019). Motor Control Changes in Low Back Pain: Divergence in Presentations and Mechanisms. J. Orthop. Sports Phys. Ther..

[12] Hides J.A., Donelson R., Lee D., Prather H., Sahrmann S.A., Hodges P.W. (2019). Convergence and Divergence of Exercise-Based Approaches That Incorporate Motor Control for the Management of Low Back Pain. J. Orthop. Sports Phys. Ther..

[13] Fourney D.R., Andersson G., Arnold P.M., Dettori J., Cahana A., Fehlings M.G., Norvell D., Samartzis D., Chapman J.R. (2011). Chronic Low Back Pain: A Heterogeneous Condition With Challenges for an Evidence-Based Approach. Spine.

[14] Foster N.E., Hill J.C., O’Sullivan P., Hancock M. (2013). Stratified models of care. Best Pract. Res. Clin. Rheumatol..

[15] O’Sullivan P. (2005). Diagnosis and classification of chronic low back pain disorders: Maladaptive movement and motor control impairments as underlying mechanism. Man. Ther..

[16] Sahrmann S.A. (2002). Diagnosis and Treatment of Movement Impairment Syndromes.

[17] McKenzie R., May S. (2003). Lumbar Spine, Mechanical Diagnosis and Therapy.

[18] Lemeunier N., Jeoun E.B., Suri M., Tuff T., Shearer H., Mior S., Wong J.J., da Silva-Oolup S., Torres P., D’Silva C. (2018). Reliability and validity of clinical tests to assess posture, pain location, and cervical spine mobility in adults with neck pain and its associated disorders: Part 4. A systematic review from the cervical assessment and diagnosis research evaluation (CADRE) collaboration. Musculoskelet. Sci. Pract..

[19] Fedorak C., Ashworth N., Marshall J., Paull H. (2003). Reliability of the Visual Assessment of Cervical and Lumbar Lordosis: How Good Are We?. Spine.

[20] Astfalck R.G., O’Sullivan P.B., Straker L.M., Smith A.J., Burnett A., Caneiro J.P., Dankaerts W. (2010). Sitting postures and trunk muscle activity in adolescents with and without nonspecific chronic low back pain: An analysis based on subclassification. Spine.

[21] Gombatto S.P., Collins D.R., Sahrmann S.A., Engsberg J.R., Van Dillen L.R. (2007). Patterns of lumbar region movement during trunk lateral bending in 2 subgroups of people with low back pain. Phys. Ther..

[22] Dankaerts W., O’Sullivan P., Burnett A., Straker L. (2006). Differences in sitting postures are associated with nonspecific chronic low back pain disorders when patients are subclassified. Spine.

[23] Hemming R., Sheeran L., van Deursen R., Sparkes V. (2018). Non-specific chronic low back pain: Differences in spinal kinematics in subgroups during functional tasks. Eur. Spine J..

[24] Sheeran L., Sparkes V., Caterson B., Busse-Morris M., van Deursen R. (2012). Spinal Position Sense and Trunk Muscle Activity During Sitting and Standing in Nonspecific Chronic Low Back Pain Classification Analysis. Spine.

[25] Don R., Capodaglio P., Cimolin V., Benedetti M.G., D’Osualdo F., Frigo C., Vismara L., Negrini S. (2012). Instrumental measures of spinal function: Is it worth? A state-of-the art from a clinical perspective. Eur. J. Phys. Rehabil. Med..

[26] Slater H., Briggs A.M. (2017). Models of Care for musculoskeletal pain conditions: Driving change to improve outcomes. Pain Manag..

[27] Al-Amri M., Nicholas K., Button K., Sparkes V., Sheeran L., Davies J.L. (2018). Inertial Measurement Units for Clinical Movement Analysis: Reliability and Concurrent Validity. Sensors.

[28] Giansanti D., Maccioni G., Benvenuti F., Macellari V. (2007). Inertial measurement units furnish accurate trunk trajectory reconstruction of the sit-to-stand manoeuvre in healthy subjects. Med. Biol. Eng. Comput..

[29] Ha T.H., Saber-Sheikh K., Moore A.P., Jones M.P. (2012). Measurement of lumbar spine range of movement and coupled motion using inertial sensors—A protocol validity study. Man. Ther..

[30] Howarth S.J., Graham R.B. (2015). Sensor positioning and experimental constraints influence estimates of local dynamic stability during repetitive spine movements. J. Biomech..

[31] Beange K., Chan A., Graham R. (2019). Wearable sensor performance for clinical motion tracking of the lumbar spine. CMBES Proc..

[32] Beange K.H., Chan A.D., Beaudette S.M., Graham R.B. (2019). Concurrent validity of a wearable IMU for objective assessments of functional movement quality and control of the lumbar spine. J. Biomech..

[33] Bauer C.M., Heimgartner M., Rast F.M., Ernst M.J., Oetiker S., Kool J. (2016). Reliability of lumbar movement dysfunction tests for chronic low back pain patients. Man. Ther..

[34] Bauer C.M., Rast F.M., Ernst M.J., Oetiker S., Meichtry A., Kool J., Rissanen S.M., Suni J.H., Kankaanpää M. (2015). Pain intensity attenuates movement control of the lumbar spine in low back pain. J. Electromyogr. Kinesiol..

[35] Bayartai M.E., Taulaniemi A., Tokola K., Vähä-Ypyä H., Parkkari J., Husu P., Kankaanpää M., Vasankari T., Michael Bauer C., Luomajoki H. (2023). Role of the interaction between lumbar kinematics and accelerometer-measured physical activity in bodily pain, physical functioning and work ability among health care workers with low back pain. J. Electromyogr. Kinesiol..

[36] Graham R.B., Dupeyron A., van Dieën J.H. (2020). Between-day reliability of IMU-derived spine control metrics in patients with low back pain. J. Biomech..

[37] Larivière C., Mecheri H., Shahvarpour A., Gagnon D., Shirazi-Adl A. (2013). Criterion validity and between-day reliability of an inertial-sensor-based trunk postural stability test during unstable sitting. J. Electromyogr. Kinesiol..

[38] Lee J., Yoon C., Kim K., Cho M., Kim H.C., Chung S.G. (2019). Lumbar Stability in Healthy Individuals and Low Back Pain Patients Quantified by Wall Plank-and-Roll Test. PM&R.

[39] Meinke A., Peters R., Knols R.H., Swanenburg J., Karlen W. (2022). Feedback on Trunk Movements from an Electronic Game to Improve Postural Balance in People with Nonspecific Low Back Pain: Pilot Randomized Controlled Trial. JMIR Serious Games.

[40] Mjøsund H.L., Boyle E., Kjaer P., Mieritz R.M., Skallgård T., Kent P. (2017). Clinically acceptable agreement between the ViMove wireless motion sensor system and the Vicon motion capture system when measuring lumbar region inclination motion in the sagittal and coronal planes. BMC Musculoskelet. Disord..

[41] Pimentel R., Potter M.N., Carollo J.J., Howell D.R., Sweeney E.A. (2020). Peak sagittal plane spine kinematics in female gymnasts with and without a history of low back pain. Clin. Biomech..

[42] Senington B., Lee R.Y., Williams J.M. (2020). Biomechanical risk factors of lower back pain in cricket fast bowlers using inertial measurement units: A prospective and retrospective investigation. BMJ Open Sport Exerc. Med..

[43] Tanigawa A., Morino S., Aoyama T., Takahashi M. (2018). Gait analysis of pregnant patients with lumbopelvic pain using inertial sensor. Gait Posture.

[44] Telles G.F., Ferreira A.S., Junior P.M.P., Lemos T., Bittencourt J.V., Nogueira L.A.C. (2022). Concurrent validity of the inertial sensors for assessment of balance control during quiet standing in patients with chronic low back pain and asymptomatic individuals. J. Med. Eng. Technol..

[45] Triantafyllou A., Papagiannis G., Stasi S., Gkrilias P., Kyriakidou M., Kampouroglou E., Skouras A.Z., Tsolakis C., Georgoudis G., Savvidou O. (2023). Lumbar Kinematics Assessment of Patients with Chronic Low Back Pain in Three Bridge Tests Using Miniaturized Sensors. Bioengineering.

[46] Trinidad-Fernández M., Beckwée D., Cuesta-Vargas A., González-Sánchez M., Moreno F.A., González-Jiménez J., Joos E., Vaes P. (2020). Validation, Reliability, and Responsiveness Outcomes Of Kinematic Assessment with an RGB-D Camera to Analyze Movement in Subacute and Chronic Low Back Pain. Sensors.

[47] Wattananon P., Kongoun S., Chohan A., Richards J. (2023). The use of statistical parametric mapping to determine altered movement patterns in people with chronic low back pain. J. Biomech..

[48] Laird R.A., Keating J.L., Ussing K., Li P., Kent P. (2019). Does movement matter in people with back pain? Investigating ‘atypical’ lumbo-pelvic kinematics in people with and without back pain using wireless movement sensors. BMC Musculoskelet. Disord..

[49] Laird R.A., Kent P., Keating J.L. (2016). How consistent are lordosis, range of movement and lumbo-pelvic rhythm in people with and without back pain?. BMC Musculoskelet. Disord..

[50] Laird R.A., Keating J.L., Kent P. (2018). Subgroups of lumbo-pelvic flexion kinematics are present in people with and without persistent low back pain. BMC Musculoskelet. Disord..

[51] Dankaerts W., O’Sullivan P., Burnett A., Straker L., Davey P., Gupta R. (2009). Discriminating healthy controls and two clinical subgroups of nonspecific chronic low back pain patients using trunk muscle activation and lumbosacral kinematics of postures and movements: A statistical classification model. Spine.

[52] Bacon Z., Hicks Y., Al-Amri M., Sheeran L. (2020). Automatic Low Back Pain Classification Using Inertial Measurement Units: A Preliminary Analysis. Procedia Comput. Sci..

[53] Ferreira-Valente M.A., Pais-Ribeiro J.L., Jensen M.P. (2011). Validity of four pain intensity rating scales. Pain.

[54] Fairbank J.C., Pynsent P.B. (2000). The Oswestry Disability Index. Spine.

[55] Roelofs J., Goubert L., Peters M.L., Vlaeyen J.W.S., Crombez G. (2004). The Tampa Scale for Kinesiophobia: Further examination of psychometric properties in patients with chronic low back pain and fibromyalgia. Eur. J. Pain.

[56] Sheeran L., Robling M. (2019). Spinal Function Assessment And Exercise Performance Framework For Low Back Pain. Orthop Procs..

[57] Waddell G., Somerville D., Henderson I., Newton M. (1992). Objective clinical evaluation of physical impairment in chronic low back pain. Spine.

[58] Mazzone B., Wood R., Gombatto S. (2016). Spine Kinematics during Prone Extension in People with and without Low Back Pain and among Classification-Specific Low Back Pain Subgroups. J. Orthop. Sports Phys. Ther..

[59] van Dijk M.J., Smorenburg N.T., Heerkens Y.F., Mollema J., Kiers H., Nijhuis-van der Sanden M.W., Visser B. (2020). Assessment instruments of movement quality in patients with non-specific low back pain: A systematic review and selection of instruments. Gait Posture.

[60] Errabity A., Calmels P., Han W.-S., Bonnaire R., Pannetier R., Convert R., Molimard J. (2023). The effect of low back pain on spine kinematics: A systematic review and meta-analysis. Clin. Biomech..

[61] Laird R.A., Gilbert J., Kent P., Keating J.L. (2014). Comparing lumbo-pelvic kinematics in people with and without back pain: A systematic review and meta-analysis. BMC Musculoskelet. Disord..

[62] Matheve T., De Baets L., Bogaerts K., Timmermans A. (2019). Lumbar range of motion in chronic low back pain is predicted by task-specific, but not by general measures of pain-related fear. Eur. J. Pain.

[63] Sadler S.G., Spink M.J., Ho A., De Jonge X.J., Chuter V.H. (2017). Restriction in lateral bending range of motion, lumbar lordosis, and hamstring flexibility predicts the development of low back pain: A systematic review of prospective cohort studies. BMC Musculoskelet. Disord..

[64] Riley S.P., Swanson B.T., Dyer E. (2019). Are movement-based classification systems more effective than therapeutic exercise or guideline based care in improving outcomes for patients with chronic low back pain? A systematic review. J. Man. Manip. Ther..

